# Influence of Lenslet Configuration on Short-Term Visual Performance in Myopia Control Spectacle Lenses

**DOI:** 10.3389/fnins.2021.667329

**Published:** 2021-05-25

**Authors:** Xue Li, Chenglu Ding, Yuhao Li, Ee Woon Lim, Yi Gao, Bruno Fermigier, Adeline Yang, Hao Chen, Jinhua Bao

**Affiliations:** ^1^Eye Hospital and School of Ophthalmology and Optometry, Wenzhou Medical University, Wenzhou, China; ^2^National Clinical Research Center for Ocular Diseases, Wenzhou, China; ^3^Wenzhou Medical University–Essilor International Research Center (WEIRC), Wenzhou, China; ^4^R&D AMERA, Essilor International, Singapore, Singapore; ^5^R&D Essilor International, Créteil, France

**Keywords:** myopia control, visual performance, visual acuity, contrast sensitivity, modulation transfer function, lenslets

## Abstract

**Purpose:** This study aimed to evaluate short-term visual performance and optical quality of three different lenslet configurations on myopia control spectacle lenses.

**Materials and Methods:** This study utilized a cross-over design. Distance visual acuity (VA) was measured in 50 myopic children; contrast sensitivity (CS) was measured in 36 myopic children. For each test, four spectacle lenses were evaluated in a random order: single-vision lens (SVL), lens with concentric rings of highly aspherical lenslets (HAL), lens with concentric rings of slightly aspherical lenslets (SAL), and lens with honeycomb configuration of spherical lenslets (HC). The modulation transfer function (MTF) and MTF area (MTFa) were used to determine optical quality. All tests were performed monocularly on the right eye with full correction.

**Results:** HAL and SAL had larger MTFa than HC. VA in lenses with lenslets was significantly reduced compared to SVL (all *p* < 0.01). The reduction in VA was worse with HC than with SAL (*p* = 0.02) and HAL (*p* = 0.03); no effect of lenslet asphericity was found (*p* > 0.05). VA changes induced by lenslets showed no correlation with spherical equivalent refraction (all *p* > 0.05) and were weakly positively associated with age for SAL (*r* = 0.36, *p* = 0.01) and HC (*r* = 0.31, *p* = 0.03), but not for HAL (*p* = 0.30). The area under the log contrast sensitivity function (AULCSF) decreased with HAL and HC (all *p* < 0.001) in all illumination levels, and AULCSF with HAL was higher than that with HC in a photopic condition (1.17 ± 0.10 vs. 1.10 ± 0.13, *p* = 0.0004). The presence of lenslets did not affect CS at 3 cycles per degree (cpd) (*p* = 0.80). At 6 to 18 cpd, CS was significantly reduced by HAL and HC (all *p* < 0.05), but not SAL (*p* > 0.05) compared to SVL. At high spatial frequencies (>12 cpd) both SAL and HAL reduced CS significantly less than HC (all *p* < 0.01).

**Conclusion:** Short-term visual performance was minimally impaired by looking through the lenslet structure of myopia control spectacle lenses. Concentric rings with aspherical lenslets had a significantly lower impact on both VA and CS than honeycomb configuration with spherical lenslets.

## Introduction

The prevalence of myopia is predicted to be 50% globally by the year 2050, with 10% being highly myopic (Fricke et al., 2018). This growing epidemic is a concern as the risk for myopia-related pathology is as high as 28.7% in the highly myopic population (Wong et al., 2018). Moreover, these pathologies can lead to vision impairment and heavy economic burdens (Zheng et al., 2013). As such, it is of public health interest to control myopia progression through efficient interventions (Wildsoet et al., 2019).

There are several optical interventions such as orthokeratology, bifocal spectacles, and multifocal contact lenses (Huang et al., 2016; Wildsoet et al., 2019) available in the clinic for myopia control. Recently, spectacle lens designs using lenslets to create a myopia control signal in the periphery, for example, the Defocus Incorporated Multiple Segments (DIMS) (Lam et al., 2019), spectacle lenses with slightly aspherical lenslets (SAL), and spectacle lenses with highly aspherical lenslets (HAL), have shown a promising myopia control effect (Bao et al., 2021). The efficacy of these spectacle lenses was comparable to orthokeratology (Li et al., 2016; Santodomingo-Rubido et al., 2017) and 0.01% atropine (Diaz-Llopis and Pinazo-Duran, 2018; Kinoshita et al., 2018). Moreover, spectacle lenses are non-invasive and safer than contact lenses or drugs.

However, spectacle lenses with lenslets face similar visual performance issues like multifocal contact lenses used for myopia control, especially in the peripheral part of the visual field. Lenses designed for myopia control were found to affect low-contrast visual acuity under low illuminance, while distant high-contrast vision acuity was rarely affected (Kang et al., 2017; Diec et al., 2018; García-Marqués et al., 2020; Lu et al., 2020). Lu et al. (2020) found that the DIMS showed no effect on visual acuity (VA) through the central clear zone but reduced VA by three optotypes in the defocus area with lenslets. Pauling et al., found that the multifocal soft contact lenses affected the low- and high-contrast VA on initial insertion and advocated that the effects on vision should be communicated when dispensing these lenses (Kang et al., 2017). However, Jennie et al. stated that visual acuity did not adequately reflect visual performance for multifocal contact lens. Contrast sensitivity (CS), in contrast, is a more sensitive measure, especially when the lens was significantly decentered (Fedtke et al., 2016). One study found worse visual performance with a higher addition power lens (Przekoracka et al., 2020), while another found no difference (Walline et al., 2020).

In normal, straight viewing conditions, children using spectacle lenses with lenslets in the lens periphery will look through the central clear zone, which has been shown to have no impact on VA (Lu et al., 2020). However, eye movements and possible position shifts of the spectacle frame make it possible for the visual axis to pass through the peripheral zone with lenslets. Thus, it is necessary to evaluate the visual performance through the lenslet zone to understand the impact of the lenslets to provide guidance for clinical practice. This study aimed to evaluate the optical quality and visual performance through various lenslet configurations and compare them with single-vision lenses (SVL) in children. VA and CS were used to evaluate the visual quality subjectively, and modulation transfer function (MTF) was used to estimate the optical property.

## Materials and Methods

### Subjects

This was a cross-over design study. For the VA test, 50 myopic children [mean age 12.7 ± 1.7 years, age range 10 to 15 years, mean spherical equivalent refraction (SER) −3.22 ± 1.57 D, SER range −6.50 to −0.38 D] participated; for the CS test, 36 myopic children (mean age 13.2 ± 1.2 years, age range 10 to 16 years, mean SER −3.20 ± 1.67 D, SER range −7.25 to −0.75 D) were enrolled. Subjects had no ocular pathology or former history of using myopia control interventions. During the experiment, each subject was fully corrected using a trial frame. Testing lenses with lenslets were added to the right eye while the left eye was occluded. All tests were performed immediately after fitting the lenses without any adaptation. This study adhered to the tenets of the Declaration of Helsinki and was approved by the Ethics Committee of the Eye Hospital of Wenzhou Medical University (no. 2019-091-K-87). Written informed consent was obtained from both children and their legal custodian before the study.

### Apparatus

**FIGURE 1:**
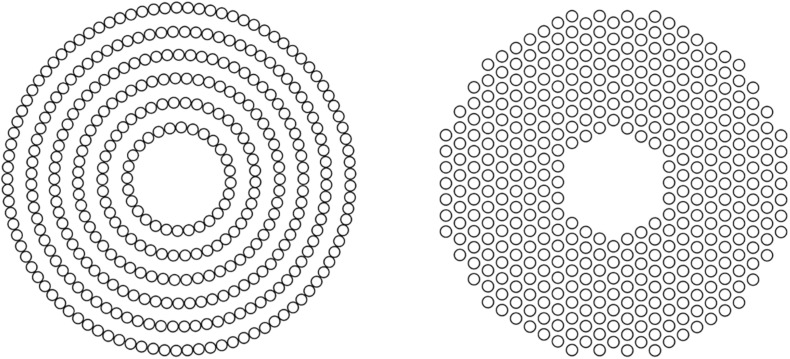
Pictorial representation of concentric rings (left) and honeycomb (right) configurations of lenslets.

All spectacle lenses were made of polycarbonate in this study. There were four designs: (1) traditional single-vision lens (SVL) as control, (2) concentric ring configuration with highly aspherical lenslets (HAL) (Figure 1, left), (3) concentric ring configuration with slightly aspherical lenslets (SAL), and (4) honeycomb configuration of spherical lenslets (HC) (Figure 1, right). For HAL and SAL, the surface of the lens without lenslets provides distance correction. The geometry of the aspheric lenslets (1.12 mm in diameter) was calculated to generate a volume of myopic defocus ranging from 1.1 to 1.9 mm (HAL) and from 1.0 to 1.3 mm (SAL) in front of the retina at any eccentricity, serving as a myopia control signal. The lenslets (1.03 mm in diameter) of HC introduce myopic defocus at a plane in front of the retina by a relative positive power (+3.50 D) (Lu et al., 2020; Zhang et al., 2020). The surface of the lens without lenslets provides distance correction. The lenslets of two configurations, concentric rings and honeycomb, provide a similar density of lenslets that was approximately 40% of the total surface area of each lens.

**FIGURE 2:**
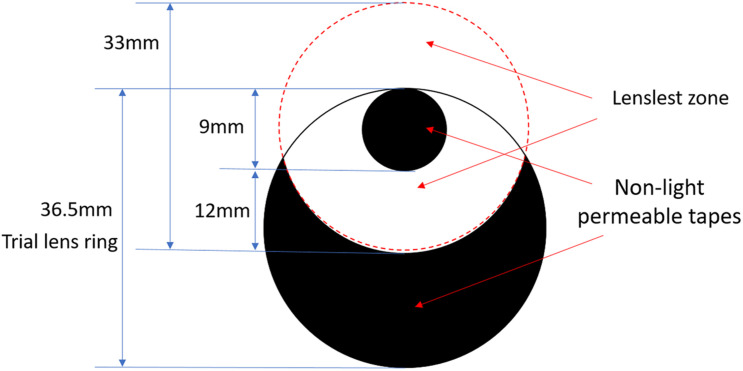
Pictorial representation of patched lenses. The large solid circle of 36.5-mm diameter represents the actual edged lens (trial lens). The small black circle of 9-mm diameter represents the patched central clear zone of the original lens, and the black crescent-shaped area represents the patched peripheral clear zone of the original lens. All four lenses were patched in identical ways.

Each of the three lenses with lenslets was mounted into a trial lens ring to maximize the lenslet zone, in which the central clear zone was on the edge of the cut lens (Figure 2). To ensure viewing only through the lenslets zone, the 9 mm of central clear zone (the small black circle) and the area beyond a distance of 12 mm from the central zone (the black crescent-shaped area) were patched by non-light-permeable tapes. The SVL was edged and covered up in the same way to ensure the same size and shape of the visual field among the lenses. During the experiments, the subjects wore a trial frame and performed the visual tests by looking through the lenslet zone. Four types of lenses were tested in random order.

### Modulation Transfer Function

Modulation transfer function (Pieh et al., 2002; Son et al., 2017) has been widely used to quantify the optical quality of the lens design, and MTFa (modulation transfer function area) can be used to predict the VA and CS outcome (Fernández et al., 2019; Armengol et al., 2020). In summary, to calculate MTF, one evaluates the complex amplitude in the pupil plane, then using fast Fourier transform (FFT) calculates the point spread function (PSF) and finally the MTF, using one center wavelength (λ = 550 nm) and assuming the pupil position directly on the glass (Voelz, 2011). The MTFa of optical simulation of three lens designs with lenslets was calculated within the spatial frequency range of 0–15 cycles per degree (cpd) (Vega et al., 2018; Jaskulski et al., 2020) on 4, 6, and 8 mm apertures by a 550-nm light source. MTFs were computed for the same pupil apertures at 5, 10, and 15 cpd.

### Contrast Sensitivity Function

**FIGURE 3:**
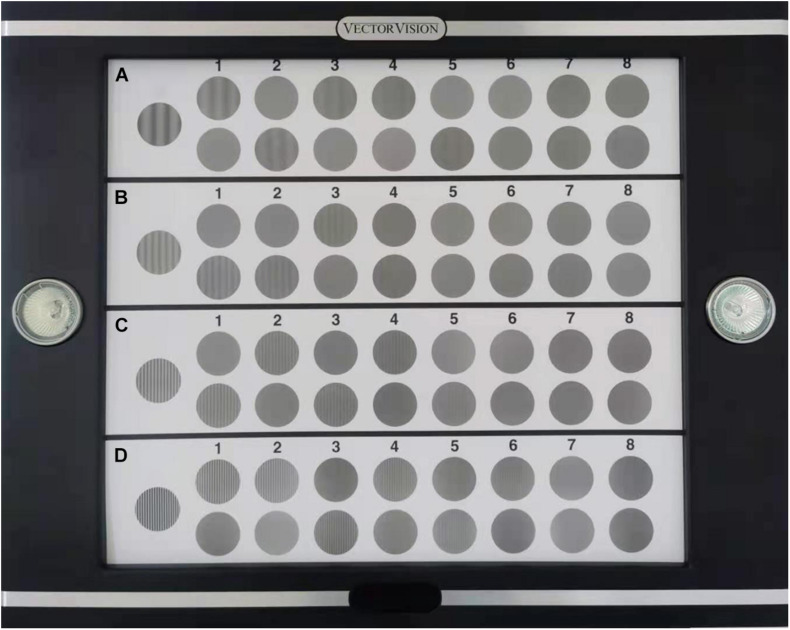
Test card of CSV-1000 for distant contrast sensitivity (CS). **(A-D)** represent four spatial frequencies, from low to high, 3, 6, 12, 18 cycles per degree (cpd).

The contrast sensitivity (CS) and glare disability with the test lenses were measured with CSV-1000 (Vector Vision Carp, United States; Figure 3). The test was performed at a distance of 2.5 m in a dark room; the translucent chart presented four spatial frequencies: 3, 6, 12, and 18 cpd, with contrast levels reduced in steps corresponding to 0.15 logCS. The testing illuminance levels from the light box included photopic (85 cd/m^2^) and mesopic (3 cd/m^2^) conditions with and without glare (Pomerance and Evans, 1994). The area under the log contrast sensitivity function (AULCSF) was calculated by summing the area under the CSF obtained from the data measured (Applegate et al., 1998) in each condition.

Before the test, two practice trials were implemented to eliminate the effect of familiarity. Then, subjects adapted to each illuminance level for 5 min before testing. The four testing lenses were applied in a random order with a short interval for approximately 1 min. The total testing lasted approximately 1 h.

### Visual Acuity

**FIGURE 4:**
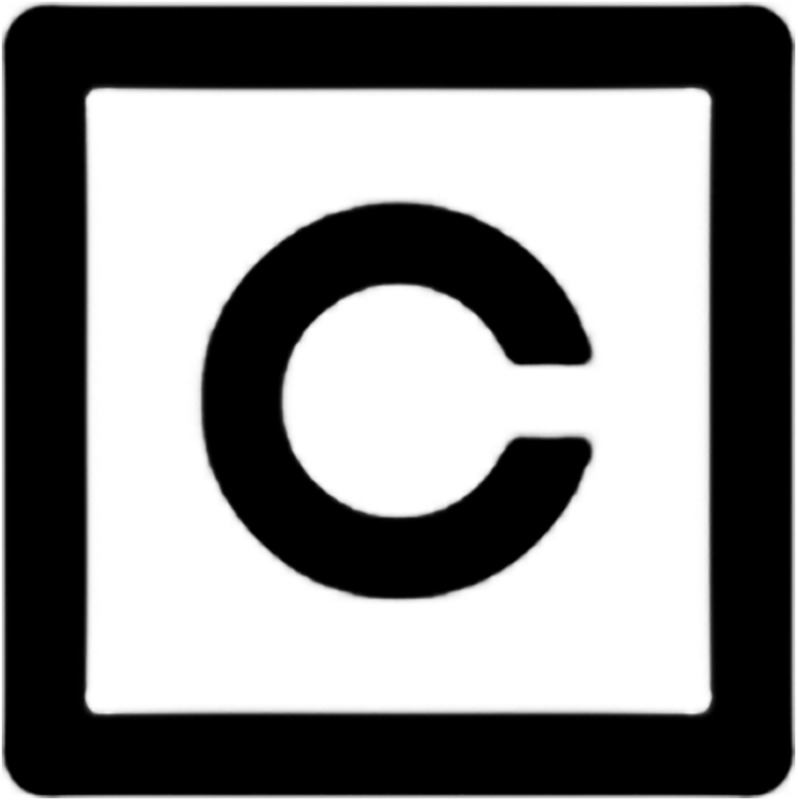
An example of the stimuli (FrACT test with high contrast, 100%).

Visual acuity was evaluated using the Freiburg Vision Test (FrACT) (Bach, 1996, 2006). Compared with the Snellen VA chart, the computerized and automated FrACT tool is free of examiner’s bias (Ma et al., 2013). A single Landolt C represented the stimulus with the opening at one of eight cardinal directions enclosed in a crowding square on a Mac screen of 21.5-in screen dimension and 1920 × 1080 resolution. The average screen luminance was 75 cd/m^2^ (Figure 4). An eight-alternative forced-choice paradigm (8-AFC) was used, in which the task was to determine the opening direction of the Landolt C among the eight possible cardinal directions (four cardinal directions and four oblique directions).

During the experiment, the testing distance was 3 m, and the illumination at the eye plane was 200 lux. Before the test, two practice trials were implemented to eliminate the effect of familiarity. Then, each of the four lenses was imposed on the right eye of the subject in a random order for testing, with a short break of 1 min in between. The total testing time was within 30 min. Landolt Cs were presented at 100% contrast, and the measurement procedure was described in detail in previous literature (Bach, 2007; Bach and Schäfer, 2016).

### Statistical Analysis

Statistical analysis was performed using the SPSS (version 25.0, SPSS, Inc.) software. Repeated-measures ANOVAs were used to test intergroup differences, if significant, followed by *post hoc* Bonferroni tests for pairwise comparisons. The statistical significance threshold was set at *p* < 0.05.

## Results

### Modulation Transfer Function

**FIGURE 5:**
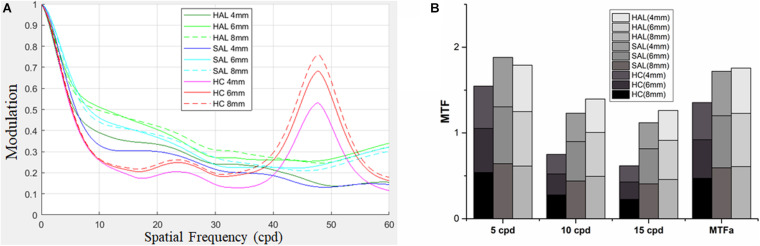
For lenses of HAL, SAL, and HC, MTFs were computed for three pupil apertures of 4, 6, and 8 mm **(A)**. MTFs at 5, 10, and 15 cpd, and MTFa from 0 up to 15 cpd at the three pupil apertures are shown in **(B)**. HAL, highly aspherical lenslets; SAL, slightly aspherical lenslets; HC, honeycomb configuration of spherical lenslets; MTF, modulation transfer function; MTFa, MTF area; cpd, cycle per degree.

A quantitative analysis of the optical performance of honeycomb and concentric ring configurations was performed using MTF and MTFa simulation through aperture sizes 4, 6, and 8 mm (Figure 5).

The MTF curves of three lenses showed similar patterns for all pupil apertures, revealing a decrease in image modulation from 0 to 20 cpd. The effect of the spherical lenslets in the honeycomb configuration was similar to that of aspherical lenslets in concentric ring configuration at low spatial frequencies (< 5 cpd). Between 5 and 35 cpd, HC decreased image modulation compared with HAL and SAL, then sharply increased it approximately 47 cpd (Figure 5A).

MTFa of honeycomb configuration was less than that of the concentric ring configurations (Figure 5B), indicating that the lenslets of concentric ring configurations would provide better optical performance.

### Contrast Sensitivity Function

**FIGURE 6:**
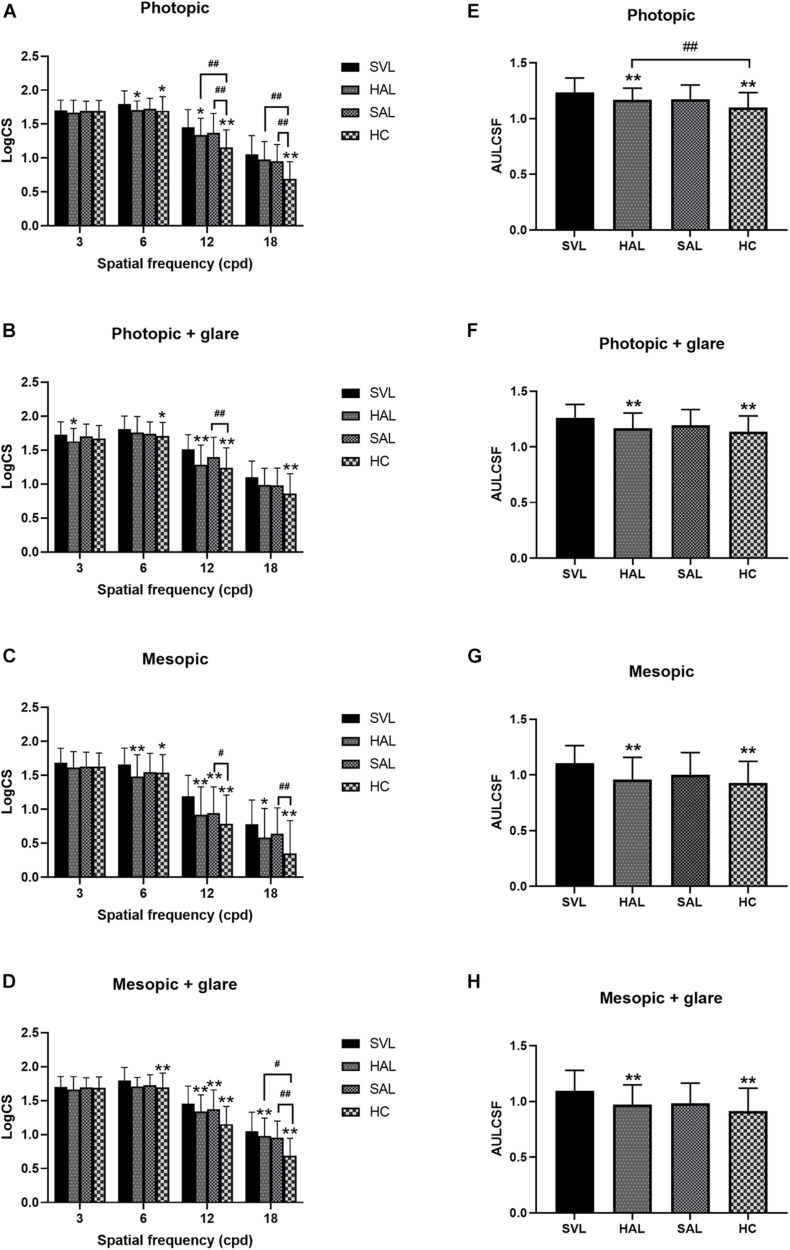
Mean log contrast sensitivity **(A–D)** and area under the log contrast sensitivity function (AULCSF) **(E–H)** with standard deviations of four tested spectacle lenses in photopic **(A,B,E,F)** and mesopic **(C,D,G,H)** conditions, with **(B,D,F,H)** and without **(A,C,E,G)** glare. (SVL for single-vision lens, HAL for spectacle lenses with concentric rings of highly aspherical lenslets, SAL for spectacle lenses with concentric rings of slightly aspherical lenslets, and HC for lenses with spherical lenslets in honeycomb configuration) *N* = 36. Asterisk (*) and number sign (#) represent significance in the Bonferroni *post hoc* test following the repeated measures ANOVA. *p < 0.05, **p < 0.01, data compared with SVL; #*p* < 0.05, ##*p* < 0.01, data compared between pairs of spectacle lenses with lenslets.

The mean AULCSF and CS values of the four tested lenses across subjects in different illuminance conditions are shown in Figure 6.

Repeated-measures ANOVAs found significant effects of lens in each condition (all *p* < 0.05). Further *post hoc* Bonferroni tests showed a pairwise difference or not between the four testing lenses. The difference between SVL and the three lenses with lenslets indicated the impact of lenslets on CS. At the low spatial frequency of 3 cpd, CS was not significantly affected by lens configurations except in the photopic condition with glare (Figure 6B), where HAL reduced CS compared to SVL. At the mid spatial frequency of 6 cpd, SAL did not significantly affect CS compared to SVL in any illuminance conditions, while HAL and HC reduced CS significantly; there was no significant difference between all the three lenslet configurations. At high spatial frequencies (12 and 18 cpd), SAL only reduced CS in the mesopic conditions (Figures 6C,D), while HAL and HC reduced CS significantly compared to SVL in most conditions. It is worth noting that HAL did not reduce CS significantly at the very high spatial frequency (SF) of 18 cpd under the photopic conditions, whereas HC did (Figures 6A,B).

Comparisons between the three lenses with lenslets found that the two concentric ring configurations HAL and SAL generated a significantly smaller impact on CS than HC at high SFs in most conditions. In the mesopic condition (Figure 6C), in contrast to the photopic condition (Figure 6A), CS at high spatial frequencies were generally reduced, and the difference between HAL and HC became less significant while SAL still showed significantly higher CS than HC. Adding glare did not reduce the general CS levels as low illuminance did, but caused the difference between the lenslet configurations at high spatial frequencies to become less significant in the photopic condition. HAL and SAL showed no significant difference in CS under any illuminance condition.

Both HAL and HC resulted in significantly lower AULCSF than SVL in all illuminance conditions (Figures 6E–H), with and without glare (all *p* < 0.001). SAL did not cause any significant change in AULCSF compared to SVL (all *p* > 0.05). Comparisons between the spectacle lenses with lenslets revealed that AULCSF of HAL was significantly higher than that of HC in the photopic condition (1.17 ± 0.10 vs. 1.10 ± 0.13, *p* = 0.0004, Figure 6E), but not in other illuminance conditions (Figures 6F–H).

### Visual Acuity

**FIGURE 7:**
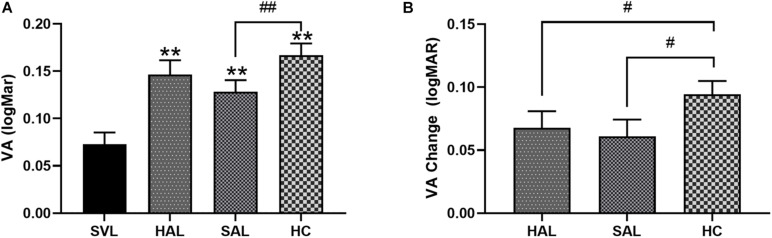
**(A)** Mean visual acuity (VA) with standard errors of four lenses (SVL for single-vision lens, HAL for spectacle lenses with concentric rings of highly aspherical lenslets, SAL for spectacle lenses with concentric rings of slightly aspherical lenslets, and HC for lenses with spherical lenslets in honeycomb configuration) and **(B)** relative VA changes from SVL of three lenses with lenslets in logMAR unit. *N* = 50. Asterisk (*) and number sign (#) represent significance in the Bonferroni *post hoc* test following the repeated measures ANOVA. **p < 0.01, comparisons of each of the three lenses with lenslets to SVL; #*p* < 0.05, ##*p* < 0.01, comparisons between each pair of the three lenses with lenslets.

The mean VA through four lenses was 0.07 ± 0.09 logMAR (SVL), 0.15 ± 0.10 logMAR (HAL), 0.13 ± 0.09 logMAR (SAL), and 0.17 ± 0.09 logMAR (HC), respectively (Figure 7A). Repeated-measures one-way ANOVA found a significant effect of lens design on VA [*F*_(__2_._8_,_134_._9__)_ = 23.52, *p* < 0.001]. *Post hoc* Bonferroni tests showed that, compared with SVL, VA in lenses with lenslets significantly decreased (all *p* < 0.001). VA through SAL was significantly higher than through HC (*p* = 0.004).

The reduction in VA caused by lenslets relative to SVL was 0.07 ± 0.09 for HAL, 0.06 ± 0.09 for SAL, and 0.09 ± 0.07 logMAR for HC, respectively. The drop in VA caused by aspherical lenslets in concentric rings was significantly less than that caused by spherical lenslets in honeycomb configuration (all *p* < 0.05). No significant difference was found between the two lenses with aspherical lenslets (*p* > 0.99, Figure 7B).

### Correlation Between VA and CS Changes and Refractive Errors and Age

**FIGURE 8:**
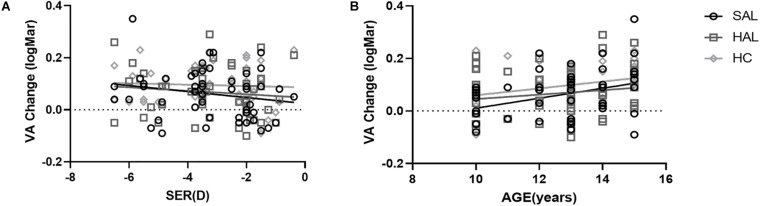
Plots of the relative changes in visual acuity (logMAR) of HAL, SAL, and HC compared to SVL as a function of SER **(A)** and age **(B)** for all subjects (*N* = 50). The lines correspond to linear regressions. Age was significantly correlated to VA changes for SAL and HC. All other correlations were not significant.

To test whether any individual factors influenced the relative reduction in VA and CS compared to SVL caused by lenslets, a correlation analysis was performed on changes in VA and CS of three lenses with lenslets and factors including the spherical equivalent refraction (SER) and age of subjects. In Figure 8, VA changes were plotted as a function of SER (Figure 8A) and age (Figure 8B) of subjects.

CS changes of all three lenses (HAL, SAL, and HC) in all spatial frequencies were not significantly correlated with age or SER in each illumination condition (all *p* > 0.05).

VA changes of HAL, SAL, and HC from SVL were not significantly correlated with SER (all *p* > 0.05, Figure 8A). Age was positively correlated with VA loss in SAL (*r* = 0.36, *y* = −0.18 + 0.02 × *x*, *p* = 0.01) and HC (*r* = 0.31, *y* = −0.07 + 0.01 × *x*, *p* = 0.03), but not in HAL (*r* = 0.16, *P* = 0.27) (Figure 8B).

## Discussion

This study presented the optical quality through simulation and short-term visual performance through clinical testing using three configurations of lenslets (HAL, SAL, and HC) on spectacle lenses that were designed for myopia control and compared them with single-vision lenses in 10–16 years old children.

We found that optical simulation could be used to predict visual performance with a spectacle lens design, and the pupil size affected the outcome. Ravikumar et al. also found that the change in VA was highly correlated with the change of MTF (Ravikumar et al., 2012). Studies on multifocal contact lenses (Kawamorita and Uozato, 2005; Madrid-Costa et al., 2012; Fernández et al., 2019) found the similar results. MTFa was also used to predict VA and CS of multifocal intraocular lenses (Vega et al., 2018; Armengol et al., 2020). In the current study, MTFs and MTFa showed that a concentric ring design impacted less visual performance than the honeycomb design. By testing VA and CS through the lenslet zones of the three spectacle lenses, we confirmed that the visual performance was aligned with the outcome of optical simulation. Moreover the CS under photopic condition was higher than that in mesopic illumination. Higher light levels induce smaller pupils by increasing the depth of focus and minimize the effects of higher-order aberrations by reducing the size of the blurred circle on the retina (Holladay et al., 1991), resulting in an increase of VA (Lombardo and Lombardo, 2010) and improved discrimination of fine stimuli (Xu et al., 2017; Mathôt and Ivanov, 2019).

Contrast sensitivity at high spatial frequencies, which reflects the ability to see fine details, was reduced by all three configurations of lenslets. The loss in CS caused by HC was significantly higher than that caused by both HAL and SAL. At both photopic and mesopic conditions, HC reduced CS at high spatial frequencies significantly more than HAL or SAL. Adding glare did not reduce the general CS as low illuminance did (Hohberger et al., 2007). Glare reduced the difference between the lenslet configuration at high spatial frequencies. CS at low spatial frequencies was not affected significantly by lenslets. The concentric rings of lenslet configuration provided better visual performance than the honeycomb configuration. Other than the configuration of lenslets, which resulted in less fragmented optics due to small aperture, the diameter of lenslets was also a factor impacting optical performance. HAL and SAL had a slightly larger lenslet diameter than HC (1.03 vs. 1.12 mm), which reduced diffraction caused by fragmented optics due to smaller aperture (Jaskulski et al., 2020).

The fact that lenslet design affects CS at high spatial frequencies suggests that lenslets also impact VA, which should be worst in HC according to MTF simulation. The results of VA tested using FrACT in the current study were consistent with the findings of CS. Jaskulski et al. also found that the DIMS decreased contrast sensitivity at high spatial frequencies (Jaskulski et al., 2020). For VA in high luminance and high contrast, HC induced the most vision loss by approximately 0.09 logMAR, followed by HAL and SAL. The lack of difference between HAL and SAL on VA in any condition indicated that the magnitude of the asphericity of the lenslets has little effect on visual performance. The loss in VA caused by aspherical lenslets in concentric rings was about half a line on a typical VA chart. However, the VA loss caused by the spherical lenslets in the honeycomb configuration was about one whole line on the VA chart. Note that the losses in CS and VA found in the current study was obtained by testing central vision through the lenslet zones. Normally, the lenslets should be located in the periphery, and central vision should be aligned with the central clear zone. Studies have found that VA was not affected when looking through the central clear zone (Lam et al., 2019; Zhang et al., 2020).

VA loss while looking through the lenslet structures was likely caused by less light focusing on the retina (Fedtke et al., 2016), similar to the simulations of MTFa with smaller aperture sizes. However, the VA changes had a positive, weak, but significant correlation with the age of subjects in SAL and HC, but not in HAL. No correlation was found between VA change and refractive error. In other words, lenslets in SAL and HC have a larger impact on visual quality in older children, but not in HAL. Although only short-term visual performance was tested in the current study, the correlation with age suggests that younger children may have an easier or faster adaptation to the lenses, which could compensate for the optical disturbance induced by lenslets while looking through the peripheral parts of the lenses. Better adaptation of blur and acceptance of the lenses were also found in younger children wearing DIMS (Lu et al., 2020) and orthokeratology lenses (Chang and Cheng, 2019) compared to their older counterparts.

Note that only short-term effect of lenslets on visual performance was tested in the current study. Any changes in VA and CS found were immediate effects without adaptation. The impact of lenses on vision often diminishes after an adaptation period. For example, multifocal soft contact lenses designed for myopia control were found to induce reduction in high-contrast VA immediately after fitting, which subsequently recovered after 2 weeks (Kang et al., 2017) or significantly improved by over 0.10 logMAR after 8 days of adaptation (Fedtke et al., 2016). However, the impact of VA may not completely disappear as was found after an adaptation period of 1 week wearing the DIMS lenses (Lu et al., 2020). However in that study, the VA through lenslets was measured with rotating eyes to different angles, rather than looking straight forward straight as in the current study. Therefore, the small impact on VA and CS on children found in the current study is likely to reduce, but persist following adaptation.

The real-life implications of slight VA and CS losses on a child’s vision are minimal. First, the measurements were performed through the lenslet zones. In the normal way of wearing spectacle lenses with lenslets, wearers look through the central clear zone that covers the visual field from zero to approximately 18° of eccentricity. The amount of time spent looking through the central clear zone will be is significantly larger than that spent in the lenslet zones. Second, the short-term loss of VA in SAL and HAL was merely approximately 0.06–0.07 logMAR, and 0.09 logMAR in HC, which were not considered clinically significant since the 95% confidence interval of repeatability of VA tests was found to be approximately 0.10 logMAR (Raasch et al., 1998) or 0.15 logMAR (Siderov and Tiu, 1999). Third, we tested only central visual performance. The impact of spectacle lenses with lenslets on the peripheral vision and performance on daily tasks in children’s life, such as reading and writing, needs further investigation.

In summary, short-term testing results on visual performance were consistent with the simulation findings. Lenslets reduced short-term visual performance manifested in lower VA and contrast sensitivity at high spatial frequencies compared with SVL. The impact varied with the characteristics and configuration of the lenslets. Spherical lenslets in the honeycomb configuration induced larger loss in VA and CS than aspherical lenslets in concentric rings. However, the level of asphericity of the lenslets showed no significant effect on visual performance. The positive correlation between the impact on VA and the subjects’ age for SAL and HC suggests better adaptation in younger children.

## Data Availability Statement

The raw data supporting the conclusions of this article will be made available by the authors, without undue reservation.

## Ethics Statement

The studies involving human participants were reviewed and approved by the Ethics Committee of the Eye Hospital of Wenzhou Medical University (no. 2019-091-K-87). Written informed consent to participate in this study was provided by the participants’ legal guardian/next of kin.

## Author Contributions

All authors listed have made substantial, direct and intellectual contribution to the work, and approved it for publication.

## Conflict of Interest

BF is employed by company R&D Essilor International, Créteil, France. JB is an Associate Director of Wenzhou Medical University–Essilor International Research Centre. YB, AY, and EWL are employees of Essilor International. This company supplied the study devices and holds the following patent applications related to this work: WO2019166653, WO2019166654, and WO2019166655. The remaining authors declare that the research was conducted in the absence of any commercial or financial relationships that could be construed as a potential conflict of interest.
